# Detection of capillary abnormalities in early diabetic retinopathy using scanning laser ophthalmoscopy and optical coherence tomography combined with adaptive optics

**DOI:** 10.1038/s41598-024-63749-7

**Published:** 2024-06-11

**Authors:** Marie Elise Wistrup Torm, Michael Pircher, Sophie Bonnin, Jesper Johannesen, Oliver Niels Klefter, Mathias Falck Schmidt, Jette Lautrup Frederiksen, Nicolas Lefaudeux, Jordi Andilla, Claudia Valdes, Pablo Loza-Alvarez, Luisa Sanchez Brea, Danilo Andrade De Jesus, Kate Grieve, Michel Paques, Michael Larsen, Kiyoko Gocho

**Affiliations:** 1 Department of Ophthalmology, Center for Research in Eye Diseases, Rigshospitalet, Section 37, Valdemar Hansens Vej 13, 2600 Glostrup, Denmark; 2 Faculty of Health and Medical Sciences, University of Copenhagen, Blegdamsvej 3B, Copenhagen N, Denmark; 3 Center for Medical Physics and Biomedical Engineering, Medical University of Vienna, Waehringer Guertel 18-20, 1090 Vienna, Austria; 4 INSERM-DGOS CIC 1423, CHNO des Quinze-Vingts, 28 Rue de Charenton, 75012 Paris, France; 5 INSERM, CNRS, Institut de La Vision, Sorbonne Université, 17 Rue Moreau, 75012 Paris, France; 6 Foundation Rothschild Hospital, 25-29, Rue Manin, 75019 Paris, France; 7 Department of Pediatrics, Herlev-Gentofte Hospital, Borgmester Ib Juuls Vej 25C, Herlev, Denmark; 8 Department of Clinical Research, Steno Diabetes Center Copenhagen, Borgmester Ib Juuls Vej 83, Herlev, Denmark; 9 Department of Neurology, Clinic of Optic Neuritis, The Danish Multiple Sclerosis Center (DMSC), Rigshospitalet, Valdemar Hansens Vej 13, Glostrup, Denmark; 10 Imagine Eyes, 18 Rue Charles de Gaulle, Orsay, France; 11 The Barcelona Institute of Science and Technology, ICFO-Institut de Ciencies Fotoniques, 08860 Castelldefels, Barcelona Spain; 12 Department of Radiology and Nuclear Medicine, Erasmus Medical Center, Dr. Molewaterplein 40, Rotterdam, The Netherlands; 13 Department of Ophthalmology, Erasmus Medical Center, Dr. Molewaterplein 40, Rotterdam, The Netherlands; 14 The Rotterdam Eye Hospital, The Rotterdam Ophthalmic Institute, Schiedamse Vest 160, Rotterdam, The Netherlands

**Keywords:** Retinal diseases, Diabetes complications

## Abstract

This study tested if a high-resolution, multi-modal, multi-scale retinal imaging instrument can provide novel information about structural abnormalities in vivo. The study examined 11 patients with very mild to moderate non-proliferative diabetic retinopathy (NPDR) and 10 healthy subjects using fundus photography, optical coherence tomography (OCT), OCT angiography (OCTA), adaptive optics scanning laser ophthalmoscopy (AO-SLO), adaptive optics OCT and OCTA (AO-OCT(A)). Of 21 eyes of 11 patients, 11 had very mild NPDR, 8 had mild NPDR, 2 had moderate NPDR, and 1 had no retinopathy. Using AO-SLO, capillary looping, inflections and dilations were detected in 8 patients with very mild or mild NPDR, and microaneurysms containing hyperreflective granular elements were visible in 9 patients with mild or moderate NPDR. Most of the abnormalities were seen to be perfused in the corresponding OCTA scans while a few capillary loops appeared to be occluded or perfused at a non-detectable flow rate, possibly because of hypoperfusion. In one patient with moderate NPDR, non-perfused capillaries, also called ghost vessels, were identified by alignment of corresponding *en face* AO-OCT and AO-OCTA images. The combination of multiple non-invasive imaging methods could identify prominent microscopic abnormalities in diabetic retinopathy earlier and more detailed than conventional fundus imaging devices.

## Introduction

Fundus photography and fluorescein angiography, together with slit-lamp biomicroscopy, established the base for the diagnosis and exploration of diabetic retinopathy^1^. This was subsequently expanded by scanning laser ophthalmoscopy (SLO)^2,3^ and optical coherence tomography (OCT)^4,5^. More recently, OCT angiography (OCTA) provided non-invasive blood flow imaging by sequential motion contrast recording, and adaptive optics (AO) enabled increased spatial resolution of several fundus imaging techniques^6^. Now, the various modalities can be combined or used in parallel to provide more comprehensive information about the posterior segment of the eye^7,8^. Specifically, AO can be combined with OCT (AO-OCT) for increased lateral resolution, to such a degree that individual cone photoreceptors can be visualized^9,10^. Moreover, the higher resolution of AO in combination with OCTA (AO-OCTA) shows individual retinal capillaries with thinner and more authentic calibers than conventional OCTA^11–13^.

Retinal capillary imaging is of particular interest for the study of diabetic retinopathy, which is the most common microvascular complication in diabetes mellitus^14–17^. Apparently, the abnormally elevated glycemia in diabetes, and perhaps the associated glycemic instability, too, lead to abnormalities in the capillary walls. As suggested by histopathologic studies, the latter initially include loss of pericytes, basement membrane thickening, loss of endothelial cells, and dilated or saccular capillaries, followed by formation of microaneurysms and small hemorrhages, and loss of capillary perfusion^18,19^. These abnormalities are mostly asymptomatic until they lead to an increased permeability of the capillary wall giving rise to edema, or ischemia giving rise to neovascularization which confers a risk of vitreous hemorrhage^20^. Microaneurysms and small hemorrhages are the first signs of diabetic retinopathy to appear on fundus photographs, while loss of pericytes, basement membrane thickening, loss of endothelial cells, dilated or saccular capillaries, and non-perfused acellular capillaries are invisible on fundus photographs^20^. Therefore, it is of interest to develop methods of in vivo imaging that can demonstrate these capillary abnormalities and non-perfusion, to clarify if they can be used as early biomarkers of diabetic retinopathy. While fluorescein angiography and OCTA can raise suspicion that capillary perfusion has been lost, they do so only by showing enlarged intercapillary spaces, the identification of which is made difficult by large variations in the anatomy of the healthy retina and its blood flow^21–23^.

The aim of the present study was to explore a prototype multi-modal retinal imaging instrument^13^ for its potential as a tool for mapping capillary irregularity and occlusion in the earliest stages of diabetic retinopathy.

## Methods

This was an observational descriptive study conducted from June 2021 to November 2022 with study participants recruited from the Steno Diabetes Center and the Rigshospitalet, both in Copenhagen, Denmark, and from the Quinze-Vingts National Ophthalmology Hospital in Paris, France.

Inclusion criteria for patient participants were having diabetes mellitus, either type 1 or type 2, without retinopathy or only mild to moderate non-proliferative diabetic retinopathy (NPDR) according to a fundus photographic evaluation using the Early Treatment of Diabetic Retinopathy Study (ETDRS) grading scale^21^. Exclusion criteria for patients were significant chronic systemic disease other than diabetes and ocular disease other than diabetic retinopathy. Exclusion criteria for healthy subjects were significant chronic systemic disease and any ocular disease.

The study was approved by the Regional Committee on Health Research Ethics (H-19068888) and the Danish Medicines Agency (journal no. 2020081671, EUDAMED CIV-ID no. CIV-20-08-034465) in Denmark, and by the Comité de Protection des Personnes (CPP) (clinical study no. 19.04.23.35149) and the Agence National de Sécurité du Médicament (ANSM) (no. IDRCB: 2019-A00942-55) in France, and conducted according to the Declaration of Helsinki. Patient participants of the French study site were recruited from a retinal imaging clinical trial (clinicaltrials.gov registration no. NCT04129021). Written informed consent was obtained from all participants. For participants below the age of 18 years, written informed consent was signed by their parents or legal guardian.

Participants underwent an eye examination consisting of assessment of best-corrected Snellen visual acuity and a minimum of procedures A or B and C plus one or more of the procedures D-J listed below. Imaging modalities as well as fundus regions of interest varied between participants depending on findings made during an initial wide-field *en face* OCT scan (C below) made with the Merlin device, a multi-modal multi-scale retinal imaging instrument with technical details published elsewhere^13^.

A. Fundus photography, ultra-widefield SLO, 200°, average wavelength 635 nm for color fundus imaging and 532 nm for fundus autofluorescence imaging at a nominal lateral resolution of 20 µm (Optos Silverstone, Dunfermline, Scotland, United Kingdom)
B. Fundus photography, ultra-widefield camera, 200°, red LED 585–640 nm, green LED 500–585 nm, blue LED 435–500 nm, and infrared laser diode 785 nm, nominal lateral resolution 7.3 µm (Clarus 500, Zeiss, Carl Zeiss AG, Oberkochen, Germany)
C. *En face* OCT, 12 × 9 mm macula block scan with 600 horizontal B-scans spaced 15 µm apart, swept-source, center wavelength 1050 nm, scan rate 200 kHz, nominal axial resolution 7 µm, nominal lateral resolution 15 µm (Merlin device^13^)
D. OCT, 9 mm horizontal transfoveal line scan and 12 × 9 mm macular and optic disc raster scan with 256 horizontal B-scans spaced 35 µm apart combined with a 12-line radial scan, swept-source, center wavelength 1050 nm, scan rate 100 kHz, nominal axial resolution 8 µm, nominal lateral resolution 20 µm (Topcon Triton, Topcon Corporation, Tokyo, Japan)
E. OCTA, 3 × 3 mm macular block scan consisting of 320 horizontal B-scans spaced 10.6 µm apart with averaging of 4 frames per B-scan (Topcon Triton, specifications in D.)
F. OCTA, 3 × 3 mm or 6 × 6 mm macular block scan, swept-source, center wavelength between 1040 and 1070 nm, scan rate 100 and 200 kHz, nominal axial resolution 6.3 µm, nominal lateral resolution ≤ 20 µm (Plex Elite, Carl Zeiss AC, Jena, Germany)
G. OCTA 12 × 9 mm or 3 × 3 mm macular block scan consisting of 450 or 400 horizontal B-scans spaced 20 or 7.5 µm apart, respectively, without averaging (Merlin device^13^)
H. AO-SLO of the inner retina, 4° × 3° consisting of 600 lines spaced 1.5 µm apart and an averaging of 12 frames per image (locations individual, see Supplementary Table S1 online). The SLO module used a superluminescent diode with a central wavelength 786 nm, and lateral resolution 20 µm (3.2 µm with adaptive optics) (Merlin device^13^)
I. AO-OCT 4° × 3° macular block scan consisting of 600 lines spaced 1.5 µm apart, nominal axial resolution 7 µm, nominal lateral resolution 4.1 µm (locations individual, see Supplementary Table S1 online) (Merlin device^13^)
J. AO-OCTA 3° × 3° macular block scan consisting of 400 lines spaced 1.13 µm apart (locations individual, see Supplementary Table S1 online) (Merlin device^13^).

### AO-SLO angiography

For a better visualization of perfused structures in the AO-SLO images (for specifications see H. above) we used an approach adapted from OCTA^24,25^. The AO-SLO images were registered to a common space by applying groupwise registration using the Elastix framework^26^. The registration procedure is formulated as an optimization problem in which a cost function $C$ is minimized with respect to the transform $T$. A Bspline transform (48 μm between control points in both x,y directions) with an Advanced Normalized Correlation cost function and Adaptive Stochastic Gradient Descent optimizer were used. The optimizer adjusts the parameters of the transform in order to minimize the difference between the images. A multi-resolution pyramid strategy with a random sampler, 5 resolutions, 128 iterations, and Bspline interpolator was used in the optimization. For each pair of AO-SLO image frames (*I*_*1,2*_*(x,y)*), a decorrelation *D*(*x,y*) was calculated:

1 $$D\left(x,y\right)=1-\frac{{I}_{1}(x,y){I}_{2}(x,y)}{\frac{1}{2}{\left({I}_{1}\left(x,y\right)\right)}^{2}+\frac{1}{2}{\left({I}_{2}\left(x,y\right)\right)}^{2}}$$

This was performed for all AO-SLO image frames recorded from the same location. The corresponding decorrelations *D(x,y)* were averaged to generate the final AO-SLO perfusion image.

The final AO-SLO image at a specific location was calculated via the average of all AO-SLO images:

2 $$I\left(x,y\right)=\frac{1}{N}\sum_{n=1}^{N}{I}_{n}$$

In order to remove noise, pixel locations that showed values in the final AO-SLO image *I (x,y)* below a certain intensity threshold were set to zero in the decorrelation image *D(x,y).*

The proposed approach (1) favors smoother changes, and it is therefore less sensitive to sharp corrections (e.g., strip-based registration can result in large corrections over a small artifact/registration error, while the constraints in the spline method prevent this), and (2) is easily reusable and has repeatable results thanks to the application of a well-known registration library (Elastix) instead of a custom-made approach. Also, it benefits from Elastix's multi-resolution scheme (we use 5 resolution levels) which ensures multi-level corrections: from coarser in the lowest resolution to finer in the highest.

This approach has been illustrated in Figs. 2E, 3D, and 4D.

## Results

**Figure 1:**
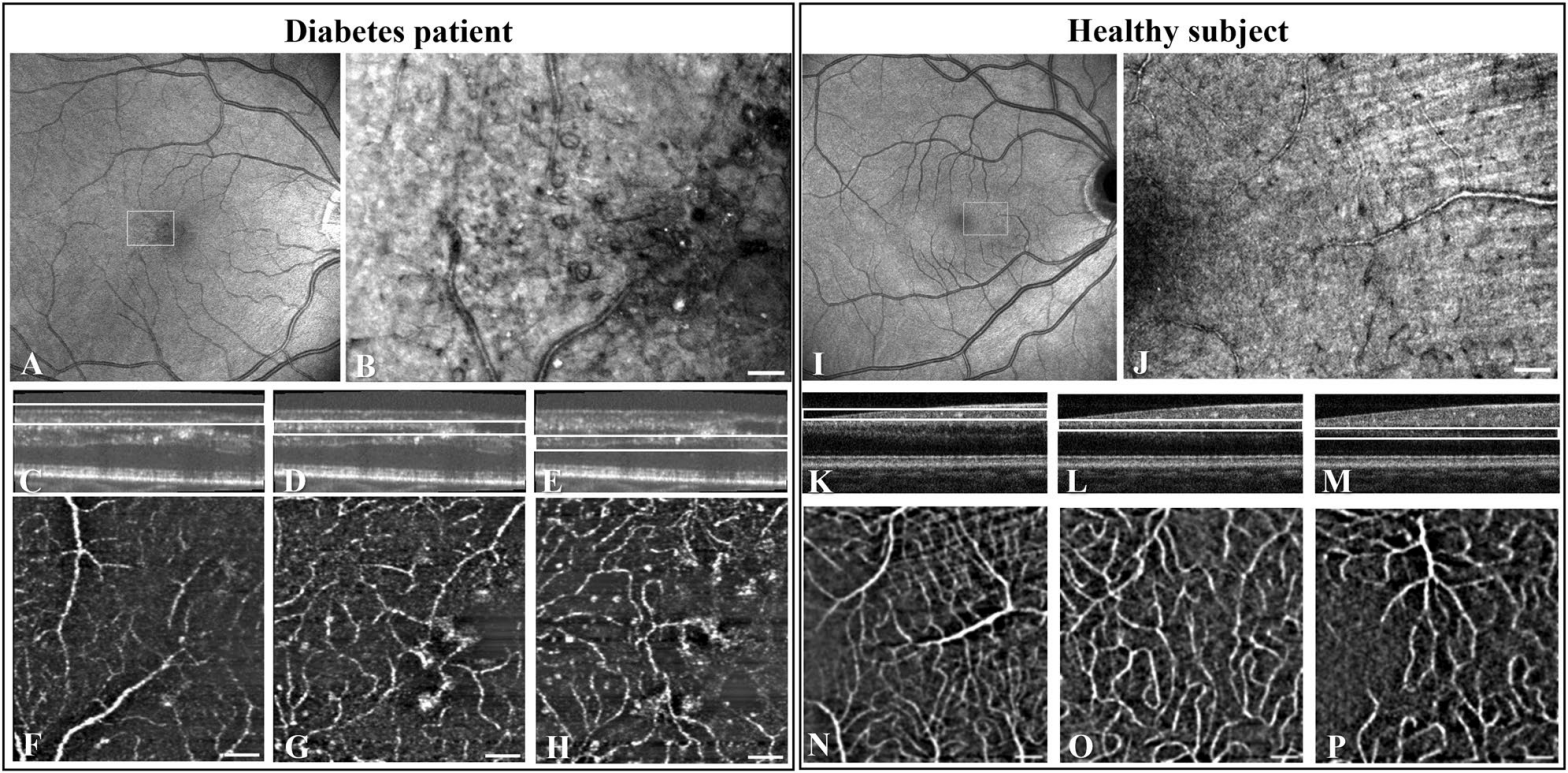
Multi-modal imaging, using the Merlin device, of a diabetes patient (participant no. 5) with moderate NPDR (**A**–**H**), compared with a healthy subject (participant no. 18) (**I**–**P**). OCT *en face* large view images (**A** and **I**) are overlayed by white rectangles corresponding to the position of the AO-SLO images (**B** and **J**, respectively) measuring 4 × 3°. Parafoveal AO-OCT B-scans are overlayed by white rectangles (**C**–**E** and **K**–**M**, respectively) indicating the segmentation slab of the overlapping AO-OCTA *en face* images measuring 3 × 3° (**F**–**H** and **N**–**P**, respectively), corresponding to three capillary plexuses: superficial (**F** and **N**), intermediate (**G** and **O**) and deep (**H** and **P**). AO-OCTA *en face* images of the intermediate and deep capillary plexuses show that compared to the healthy subject, the capillaries of the patient with diabetes appeared to be wider, more tortuous, with saccular expansions, and more irregularly spaced with larger intercapillary voids, where capillary perfusion appeared to have been lost. Scale bars represent 100 microns. See Fig. 7 for AO-OCT scans overlapping with (**G**) and (**O**).

**Figure 2:**
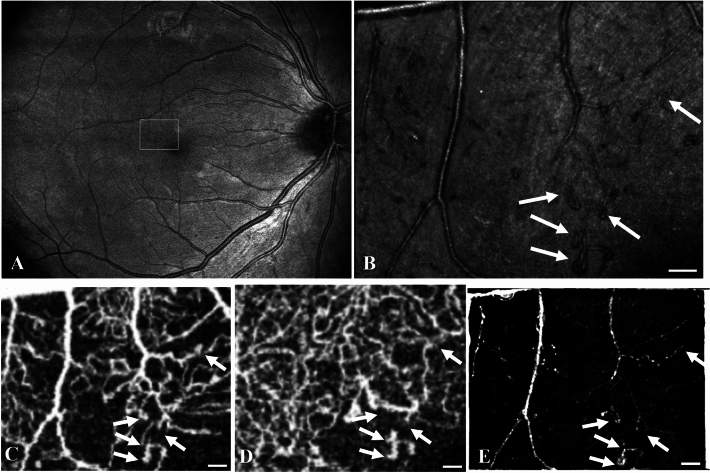
Multi-modal imaging of capillary loops in a diabetes patient with mild NPDR (participant no. 1). OCT *en face* image (**A**) overlayed by a white rectangle located 1.9° temporally and 1.6° superiorly from the foveal center indicating the location of a higher magnification AO-SLO image (**B**), both acquired with the Merlin device. The AO-SLO image shows distended capillary loops (arrows). Overlapping Topcon Triton OCTAs (superficial capillary plexus **C**, deep plexus **D**) from the same location indicates that some loops are perfused, whereas others are either non-perfused or not detectably so, or perhaps they are bridging the two plexuses and therefore split by the segmentation algorithm. A post-processed angiogram based on the Merlin AO-SLO (**E**) shows multiple perfused loops. Scale bars represent 100 microns.

**Figure 3:**
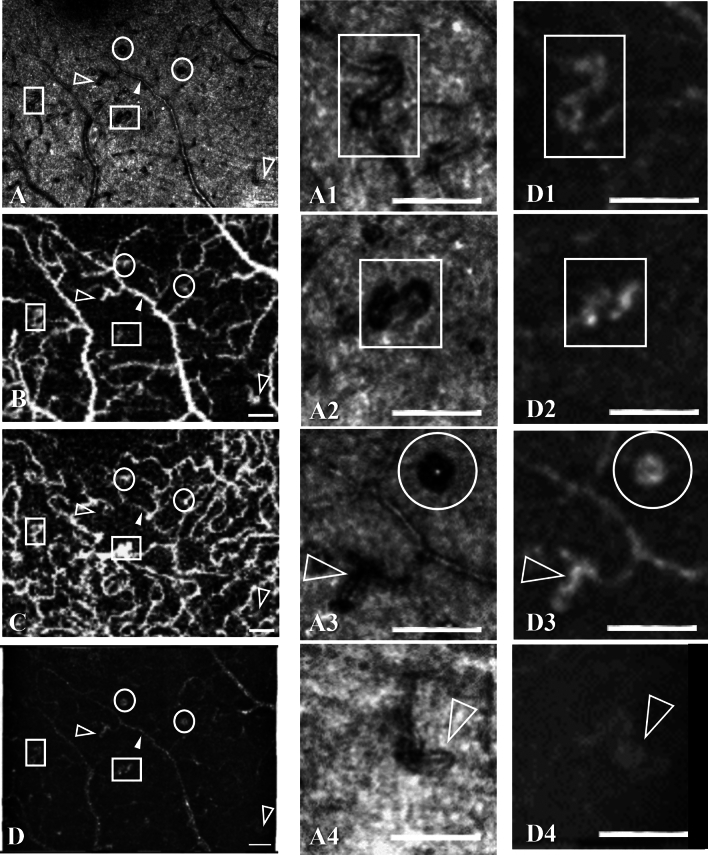
Capillary abnormalities. AO-SLO image made by the Merlin device (**A**) from which four areas have been enlarged (**A1**–**A4**), OCTA of the superficial plexus (**B**) and OCTA of the deep plexus (**C**) made by Topcon Triton, and AO-SLO angiography made by the Merlin device (**D**) from which corresponding four areas as from the AO-SLO image have been enlarged (**D1**–**D4**), all from the same area below the fovea in the same eye as in Fig. 2 (participant no. 1), showing capillary tortuosity (saturated white arrows in **A**–**D**), s-shaped inflections (white rectangles in **A**, **A1**, **A2**, **D1**, **D2**), dilation and loops (hollow white arrows in **A**, **A3**, **A4**, **D3**, **D4**), and small microaneurysms (white circles in **A**–**D**, **A3**, **D3**). In the Merlin AO-SLO angiography image (**D**), the findings are seen to be perfused. The corresponding Topcon Triton OCTA shows dilated capillary tangles in the superficial capillary plexus (**B**) and the deep plexus (**C**, white rectangles) corresponding to the aforementioned s-shaped inflections, while tortuous and dilated capillaries are visible only in the superficial plexus (hollow white arrows). Scale bars represent 100 microns.

**Figure 4:**
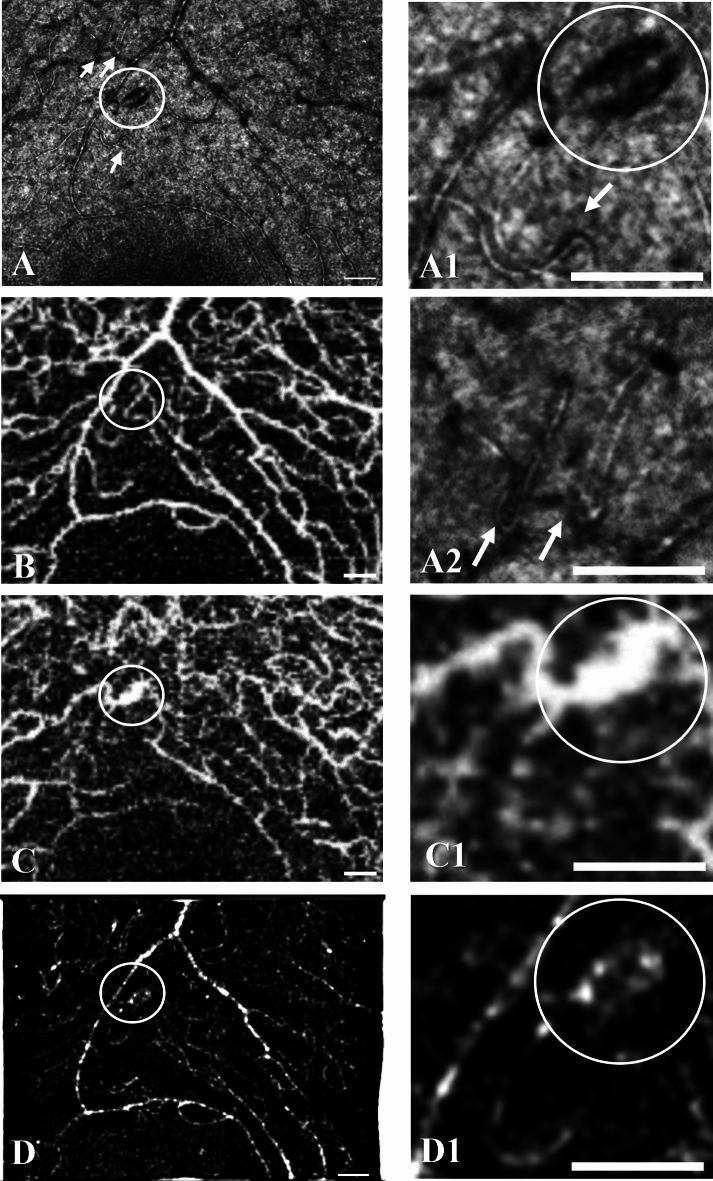
A large microaneurysm (circle) in a case of mild NPDR (participant no. 2) is seen on AO-SLO of the Merlin device (**A**, enlarged in **A1**). Capillary loops are indicated by arrows (**A**, enlarged in **A1** and **A2**). On corresponding Topcon Triton OCTA scans of the superficial capillary plexus (**B**) and of the deep capillary plexus (**C**), the microaneurysm is seen in the deep capillary plexus (**C**, enlarged in **C1**). The microaneurysm is also seen to be perfused in the post-processed AO-SLO angiography image of the Merlin device (**D**, enlarged in **D1**). Scale bars represent 100 microns.

**Figure 5:**
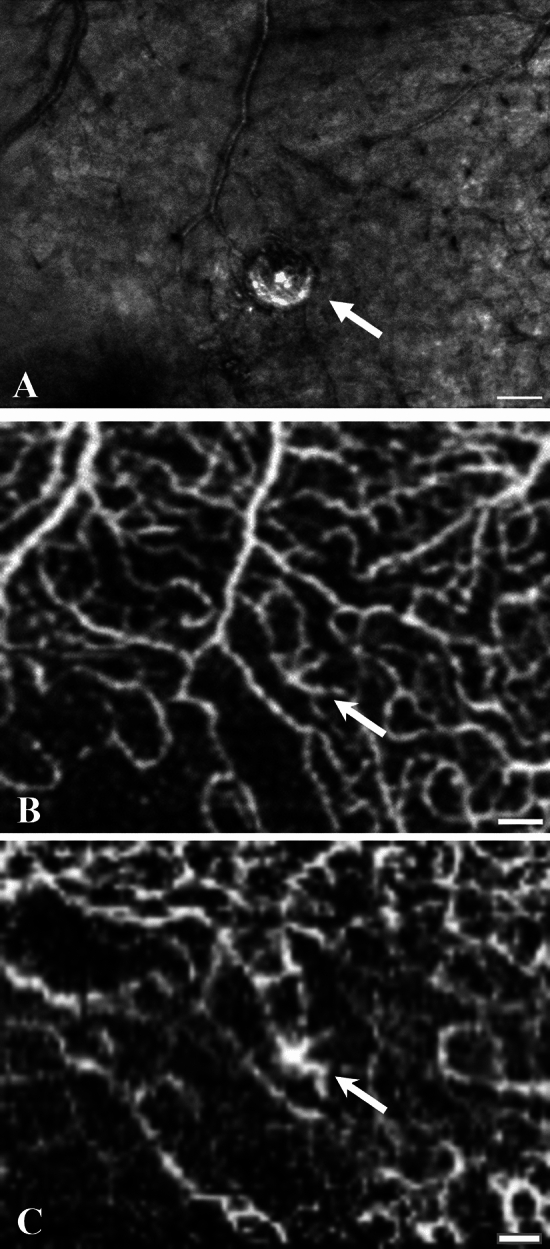
A large solitary juxtafoveal microaneurysm in a case of mild NPDR (participant no. 3) seen on AO-SLO of the Merlin device (**A**) appears on Topcon Triton OCTA to be fed and drained by a confluence of capillaries in the superficial (**B**) and deep (**C**) capillary plexuses. Scale bars represent 100 microns.

**Figure 6:**
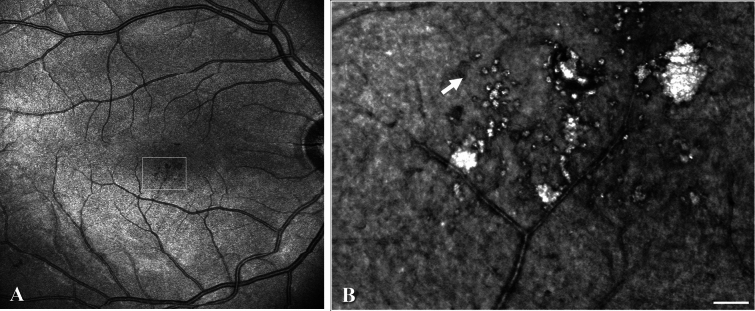
Two large microaneurysms with hyperreflective globules of presumably hard exudate seen on juxtafoveal *en face* OCT (**A**) and AO-SLO (**B**), both from the Merlin device, in a patient with mild NPDR (participant no. 4), plus a capillary loop (white arrow). Scale bar represents 100 microns.

**Figure 7:**
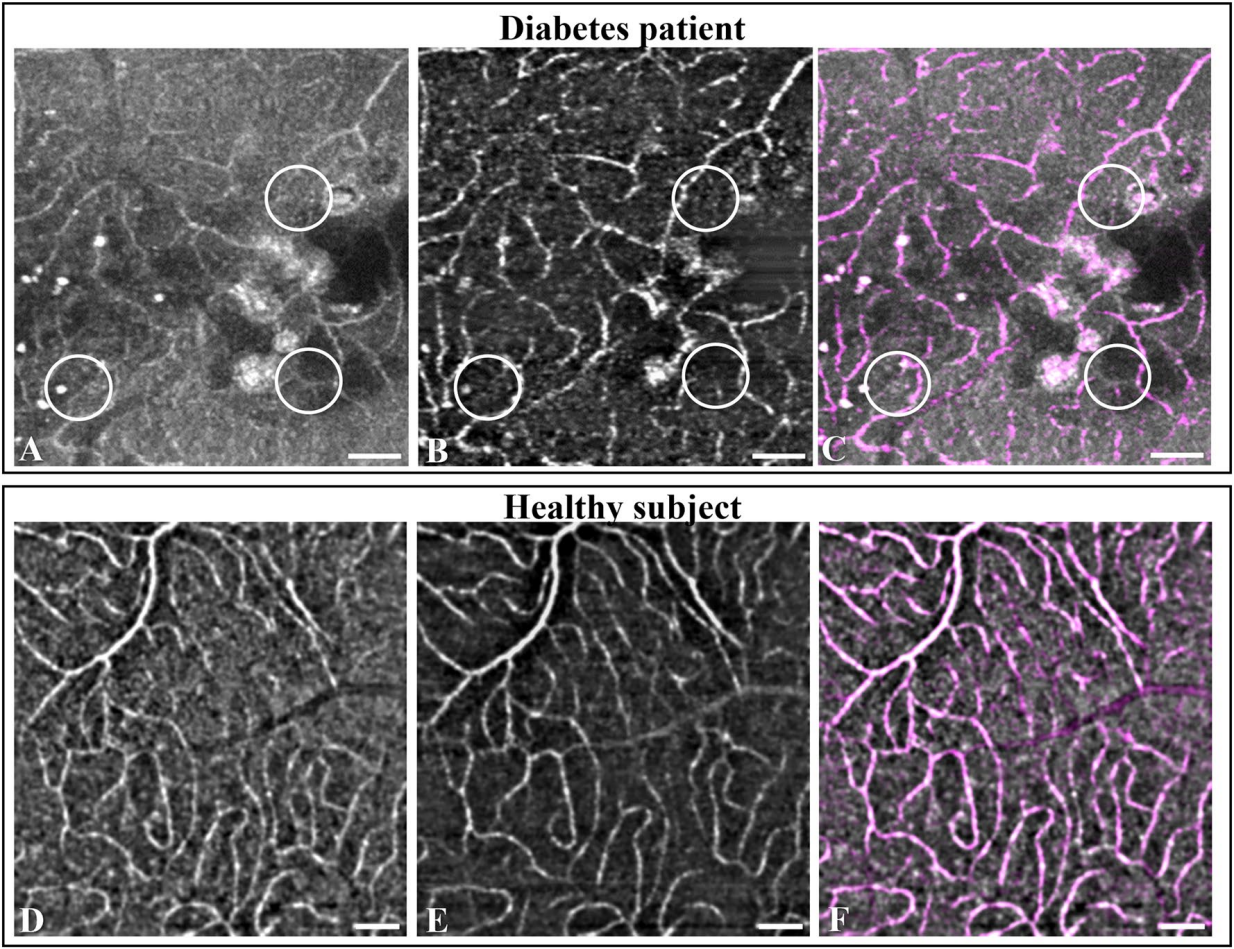
Identification and imaging of non-perfused capillaries. Alignment of *en face* AO-OCT and AO-OCTA Merlin device images of the retinal intermediate capillary plexus of a patient with diabetic retinopathy (participant no. 5) (**A**–**C**) shows three narrow strings in the AO-OCT image (**A**, encircled) that have no signal on the corresponding AO-OCTA image (**B**, encircled), a flow deficit that is highlighted in a composite rendition (**C**) where magenta indicates perfusion and gray non-perfusion. Capillaries without perfusion are not seen in the healthy subject (participant no. 18) (**D**–**F**). Scale bars represent 100 microns.

The study examined 21 eyes of 11 patients with mild to moderate NPDR and 13 eyes of 10 healthy subjects (Table 1). Imaging modalities used for each individual participant are listed in Table 2, of which the image areas of AO-SLO, AO-OCT and AO-OCTA are specified in the Supplementary Table S1 online. The presence of distinct retinal capillary abnormalities in the diabetes patients are summarized in Table 3. The findings are illustrated by images from selected cases (Figs. 1, 2, 3, 4, 5, 6, 7). No capillary abnormalities were observed in healthy subjects.

**Table 1:** Clinical characteristics of participants.

Participant no.	D/H	Age (years)	Gen﻿der	Study eye	Diabetes type	Diabetes duration (years)	HbA_1c_ (mmol/mol)	Diabetic retinopathy ETDRS stage	Visual acuity OD/OS (Snellen)
1	D	27	M	OU	1	10	80	Mild NPDR OU	1.25/1.25
2	D	30	M	OU	1	16	48	Very mild NPDR OU	1.25/1.25
3	D	56	F	OU	2	17	41	Mild NPDR OU	1.00/1.25
4	D	32	M	OD	1	4	55	Mild NPDR OD	1.00/1.00
5	D	63	F	OU	1	18	60	Moderate NPDR OU	1.00/1.00
6	D	29	F	OU	1	16	53	No DR OD, very mild NPDR OS	1.00/1.00
7	D	33	M	OU	1	18	68	Mild NPDR OD, very mild NPDR OS	1.25/1.25
8	D	31	F	OU	1	16	52	Mild NPDR OD, very mild NPDR OS	1.25/1.25
9	D	22	M	OU	1	14	47	Very mild NPDR OU	1.25/1.25
10	D	49	M	OU	2	1	57	Very mild NPDR OU	1.00/0.80
11	D	24	F	OU	1	14	55	Very mild NPDR OU	1.25/1.00
12	H	29	F	OD	–	–	–	No retinopathy	1.25/1.25
13	H	29	M	OD	–	–	–	No retinopathy	1.00/1.00
14	H	15	M	OD	–	–	–	No retinopathy	1.00/1.00
15	H	12	M	OD	–	–	–	No retinopathy	1.00/1.00
16	H	28	F	OU	–	–	–	No retinopathy	1.00/1.00
17	H	27	F	OU	–	–	–	No retinopathy	1.00/1.00
18	H	40	M	OD	–	–	–	No retinopathy	1.25/1.25
19	H	38	F	OS	–	–	–	No retinopathy	1.00/1.00
20	H	59	M	OD	–	–	–	No retinopathy	1.00/1.00
21	H	36	F	OU	–	–	–	No retinopathy	1.00/1.00

D: diabetes patient; H: healthy subject; HbA_1c_: average blood glucose level for the past three months; OD: right eye; OS: left eye; OU: both eyes; ETDRS: Early Treatment Diabetic Retinopathy Study; DK: Denmark; Fr.: France; –: not applicable.

**Table 2:** Imaging modalities (A–J specified in “Methods”) used for the participants in Table 1.

Participant	Fundus imaging	OCT	OCTA	AO-SLO	AO-OCT	AO-OCTA
1	A	C + D	E + G	H	I	J
2	A	C + D	E + G	H	–	J
3	A	C + D	E	H	I	–
4	B	C	F + G	H	I	J
5	B	C	F + G	H	I	J
6	A	C + D	E + G	H	I	J
7	A	C + D	E + G	H	I	J
8	A	C + D	E + G	H	–	J
9	A	C + D	E + G	H	I	J
10	B	C	F + G	H	I	J
11	B	C	F + G	H	I	J
12	A	C	G	H	I	J
13	A	C	G	H	I	J
14	A	C	G	H	I	-
15	A	C	G	H	I	-
16	A	C	G	H	–	J
17	A	C	G	H	–	J
18	B	C	G	H	–	J
19	B	C	G	H	–	J
20	B	C	G	H	–	J
21	B	C	G	H	–	J

OCT: optical coherence tomography, OCTA: optical coherence tomography angiography, AO: adaptive optics, SLO: scanning laser ophthalmoscopy. –: not used. H-J image locations are specified in the Supplementary Table S1 online.

**Table 3:** Retinal abnormalities of diabetes patients visualized using AO-SLO.

Participant no.	Capillary loops	Dilated or saccular capillaries	Capillary microaneurysms
1	X	X	X
2	X		X
3			X
4	X	X	X
5			X
6			
7	X		X
8	X		X
9	X		
10	X	X	X
11	X	X	X

### Capillary non-perfusion areas

Focal areas of non-perfusion of the retinal capillary plexuses were seen in the diabetes patients using OCTA or AO-OCTA. In Fig. 1, the latter method is illustrated by comparing a patient with moderate NPDR (participant no. 5) with a healthy subject (participant no. 18). The capillaries were seen to be of a more homogenous and regular spacing in the healthy subject than in the diabetes patient. The non-perfused areas in the diabetes patient were located near capillary abnormalities, the characteristics of which could be seen using AO-SLO as exemplified in the following.

### Capillary looping, dilation and tortuosity

A variety of capillary abnormalities was observed with AO-SLO in the diabetes patients, including capillary loops (participant no. 1, 2, 4, 7, 8, 9, 10 and 11), capillary tortuosity, capillary inflections, and dilated or saccular capillaries (participant no. 1, 4, 10 and 11). Most of the abnormalities could be identified on OCTA of the superficial and deep capillary plexus, although their structural characteristics did not appear as detailed as in AO-SLO, indicating they were perfused.

Capillary loops were sharply outlined by AO-SLO in a 27-year-old male with type 1 diabetes for 10 years (participant no. 1), who presented with mild NPDR in his right eye (Figs. 2 and 3) and moderate NPDR in his left eye. His previous glycemic control had been out of the normal range and so his current HbA_1c_ was 80 mmol/mol. The loops were invisible in color fundus photographs and less conspicuous or absent in OCTA, where imaging was occasionally split by the separate rendition of the superficial and deep capillary plexuses (Fig. 2). A post-processed motion-contrast based rendition of the AO-SLO image showed distinct perfusion of a large fraction of the loops and narrower vessels (Fig. 2).

In the same eye, AO-SLO imaging inferior of the foveal avascular zone (Fig. 3) showed capillary tortuosity, dilation, looping, s-shaped inflections, and small microaneurysms. Additionally, there were small bright dots, possibly representing an early stage of hard exudate formations. In the corresponding OCTA scans, capillary loops in the superficial plexus were seen to be perfused. An s-shaped capillary inflection to the left in the AO-SLO image appeared to be perfused in both plexuses, whereas another one in the middle of the field appeared to be perfused mainly in the deep capillary plexus on OCTA. Microaneurysms seen on OCTA were often small and might easily have been missed, had only OCTA been used without AO-SLO. Notice also the non-perfusion near the capillary abnormalities on OCTA.

### Capillary microaneurysms

Microaneurysms were seen on AO-SLO as distended hyporeflective extensions of capillaries with surrounding assemblies of hyperreflective granularities of varying size and reflectivity (participant no. 1, 2, 3, 4, 5, 7, 8, 10, 11).

An elongated microaneurysm, capillary loops, and abnormally large capillaries at the edge of the foveal avascular zone were seen on AO-SLO (Fig. 4) in a 30-year-old male with type 1 diabetes of 15 years’ duration (participant no. 2) and mild NPDR in both eyes. His previous metabolic control was good as was his current HbA_1c_ of 48 mmol/mol (the upper value of the normal HbA_1c_ range). A hyperreflective line along the axis of the microaneurysm on AO-SLO may represent a perfused center. The corresponding OCTA showed that the microaneurysm was located in and likely drained by the deep capillary plexus, whereas the feeding arteriolar capillary may have been in the superficial plexus (Fig. 4).

A solitary microaneurysm juxtafoveal in each eye of a 56-year-old female with type 2 diabetes for 17 years (participant no. 3) presenting with mild NPDR in both eyes, prior good metabolic control and a current HbA_1c_ of 41 mmol/mol, were seen on color fundus photographs. AO-SLO imaging of the microaneurysm on the right eye showed that it had a central hyperreflectivity, consistent with a high rate of perfusion (Fig. 5). In the corresponding OCTA, the microaneurysm was located mainly in the deep capillary plexus while it appeared to be connected to an arteriole of the superficial plexus (Fig. 5).

Juxtafoveal microaneurysms were also seen using AO-SLO in the right eye of a 32-year-old male with type 1 diabetes for 4 years (participant no. 4) presenting with mild NPDR in both eyes and macular edema in the right eye, and a current HbA_1c_ of 55 mmol/mol (Fig. 6). The microaneurysms contained hyperreflective patches suggestive of flow being maintained despite the blood having to pass through the microaneurysm^27^. Additionally, clusters of small, round hyperreflective elements may represent hard exudates.

### Non-perfused capillaries

Direct visualization of non-perfused capillaries, also called ghost vessels, was made by overlaying an AO-OCT *en face* image on a corresponding angiographic AO-OCTA *en face* image. This is illustrated by analysis of AO-OCT and AO-OCTA block scans of a 63-year-old female with type 1 diabetes (participant no. 5) presenting with moderate NPDR, macular edema and hard exudates. Alignment of two sets of respective *en face* images of the intermediate capillary plexus of a temporal juxtafoveal area of her right eye enabled identification of non-perfused capillaries, so-called “ghost vessels”: Three abnormally narrow hyperreflective strings in the AO-OCT reflectivity map, two oriented near-horizontally and one diagonally, connecting adjacent strings of normal caliber, showed no sign of perfusion in the corresponding motion-contrast based AO-OCTA map (Fig. 7). Thus, non-perfused capillaries were recognizable from their presence on AO-OCT combined with their absence on AO-OCTA. In a 40-year-old healthy subject (participant no. 18), a similar image analysis showed perfusion of all the retinal capillaries (Fig. 7).

## Discussion

This study used the non-invasive retinal imaging methods, SLO, OCT and OCTA, in combination with AO assistance, in patients with early diabetic retinopathy and healthy subjects. It demonstrated that AO-SLO can show capillary loops and focal capillary dilation in mild NPDR, lesions that only show up as red dots on a conventional fundus photograph, if they can be seen at all. Conventional OCTA scans or motion contrast analysis of consecutive AO-SLO frames could be used to indicate if the abnormalities were perfused or not. Furthermore, comparing high-resolution AO-OCT and AO-OCTA images and identifying lack of motion contrast signal in the latter corresponding to capillaries in the former enabled direct identification of capillary non-perfusion, a characteristic that is otherwise only indirectly identified as lack of motion contrast signal in OCTA scans. No such abnormalities were observed in healthy subjects.

Capillary dilation, looping, occlusion and non-perfusion using AO-SLO have previously been reported in longitudinal case studies of severe NPDR and proliferative diabetic retinopathy (PDR)^16,17^. Capillary loops were interpreted as precursors of or components of intraretinal microvascular abnormalities (IRMAs), a broad descriptive term mainly covering small arteriovenous shunt vessels characteristic of severe NPDR^16,17^. Our findings of capillary loops in mild NPDR suggest that the IRMA term can be refined and advanced to earlier stages of disease by using improved resolution and contrast of a new generation of fundus imaging devices.

Previous analysis of structural characteristics of microaneurysms using AO-SLO^15,17^ in different stages of diabetic retinopathy has described intraluminal hyperreflective structures as in our study, and the method has been reported to have a higher sensitivity for the detection of microaneurysms than fundus photography^15^. We propose that supplemental analysis of the reflection patterns on OCT B-scans can be used to assess their degree of perfusion^27–29^. The subject is complex because retinal hemodynamics vary with glycemia^30^.

Furthermore, blood flow velocity may be determined using a post-acquisitional software analysis of images from an AO-SLO video based on the angle by which erythrocytes have moved between consecutive images^22^. Using this, prior studies have found higher blood flow variation in diabetes patients with NPDR and without retinopathy than in healthy subjects^22^, and higher blood flow velocity in diabetes patients without retinopathy than in NPDR^14^. Another analysis of AO-SLO videos acquired at different time points has also been used to detect transient non-perfusion^31,32^. The prototype used in this study was not capable of performing AO-SLO videos.

Histopathology studies may help interpret our findings. Early abnormalities in diabetic retinopathy include basement membrane thickening, loss of pericytes and endothelial cells, and the followed accumulation of material inside the vascular lumen, such as invading glial cells, endothelial cell debris, pericyte ghosts, blood cells and thromboembolic material^33^. These abnormalities are accompanied by capillary dilation, looping and irregularity, which appear to cause decreased blood flow, acellular capillaries, and capillary occlusion. Capillary loops have been described as varicose in shape and mainly located on the venous side on the border to avascular zones. Venous loops, large enough to be recognized in fundus photographs in patients with PDR, have by immunohistochemistry been suggested to be the basis for endothelial cell proliferation within the vascular walls as well as neovascularization, as a reaction to ischemia^34^.

Trypsin digest studies of the human retina have shown that such abnormalities are associated with the microaneurysms that are the prototype early ophthalmoscopically visible lesion in diabetic retinopathy^18,19^. Thus, the capillary aneurysmal lesions seen using the trypsin digest technique^18^, which appear as the s-shaped capillary inflections we found using AO-SLO, have been suggested to be derived from capillary loops. Retinal microaneurysms have furthermore been examined with electron microscopy and confocal scanning laser microscopy altogether suggesting that their intraluminal content, which we saw as hyperreflective granularities in AO-SLO imaging, may represent aggregates of endothelial cells^18,35^, inflammatory cells, erythrocytes and lipid deposits^35,36^. Because microaneurysms can be confused with dot hemorrhages on fundus photographs and because retinal hemorrhages are evanescent, better methods for detection of diabetic retinopathy may help improve the management of diabetes^18^.

The present study used a protype (Merlin device) that combines modalities and has a potential for routine clinical implementation. In comparison with Triton and RTX1 devices the Merlin device has a similar footprint size: Merlin footprint ~ 50 × 50 × 60 cm (W × D × H) and Triton footprint ~ 35 × 55 × 55 cm (W × D × H). In terms of internal complexity, the Merlin device is notably more complex than Triton because of the implementation of both AO, pupil tracking and retinal tracking. The overall imaging time using the Merlin device was slightly longer, mostly because the smaller field of view in AO-mode required acquisitions at various locations and focus settings. Nevertheless, the opportunity to select and refind a specific fundus area enabled detailed imaging and follow-up.

The combination of methods used in this study could be valuable in the early and precise detection of retinopathy and the close monitoring of retinopathy progression or regression, especially by moving from suspicion of capillary perfusion loss to detailed positive identification of such loss^37^. In vivo correlation of structural and angiographic OCT for positive identification of capillary non-perfusion has only, to the best of our knowledge, been done in a manual analysis of quiescent diabetic retinopathy^38^. This will be of particular value in early stages of diabetic retinopathy, providing an opportunity for optimizing early diabetes treatment and for defining an early endpoint for clinical trials.

This study was limited by its exploratory nature and the small sample size. Blood flow at rates that fell outside the motion contrast detection range of the OCTA or AO-OCTA modalities may have been overlooked^25^. Also, comparing AO-OCT and AO-OCTA images reveals that non﻿–perfused capillaries appear less distinct than perfused ones. One possible explanation might be that these capillaries contain fewer and most likely deformed erythrocytes. Erythrocytes are known to be highly backscattering and represent the major contrast mechanism of capillaries in OCT. Some angiographic signals in AO-OCTA were sometimes not corresponding to a structural capillary in the AO-OCT. This points to the fact that vessel contrast in AO-OCT, and for that matter also in AO-SLO images, is complicated. This may lead to an overinterpretation of observed differences. Other methodological limitations, such as minor movement artefacts, may have influenced our results. Fluorescein angiography was not used, partly because it only shows the inner retinal capillary layer and partly because the technique is invasive requiring an intravenous contrast agent.

The crude incidence and the severity of diabetic retinopathy are increasing globally, although the need for treatment of diabetic retinopathy is decreasing in populations with access to modern diabetes care^39^. In both contexts, however, earlier and more detailed monitoring of the earliest signs of capillary abnormalities in retinopathy may be valuable for the titration of diabetes care and the avoidance of long-term complications of diabetes. Such monitoring could be enabled by combination of sensitive methods as used in the present study.

### Supplementary Information

Supplementary Table S1.

## Data Availability

The data generated and/or analyzed during the current study are available from the corresponding author on reasonable request provided that a data processing agreement is completed and fulfilled.

## References

[1] Abramoff MD, Garvin MK, Sonka M (2010). Retinal imaging and image analysis. IEEE Rev. Biomed. Eng..

[2] Webb RH, Hughes GW (1981). Scanning laser ophthalmoscope. IEEE Trans. Biomed. Eng..

[3] Aumann S, Donner S, Fischer J, Müller F (2019). High Resolution Imaging in Microscopy and Ophthalmology: New Frontiers in Biomedical Optics.

[4] Fujimoto JG (2003). Optical coherence tomography for ultrahigh resolution in vivo imaging. Nat. Biotechnol..

[5] Fercher AF, Drexler W, Hitzenberger CK, Lasser T (2003). Optical coherence tomography—Principles and applications. Rep. Prog. Phys..

[6] Burns SA, Elsner AE, Sapoznik KA, Warner RL, Gast TJ (2019). Adaptive optics imaging of the human retina. Prog. Retin. Eye Res..

[7] Roorda A (2002). Adaptive optics scanning laser ophthalmoscopy. Opt. Express.

[8] Burns SA, Marcos S, Elsner AE, Bara S (2002). Contrast improvement of confocal retinal imaging by use of phase-correcting plates. Opt. Lett..

[9] Jonnal RS (2016). A review of adaptive optics optical coherence tomography: Technical advances, scientific applications, and the future. Investig. Ophthalmol. Vis. Sci..

[10] Pircher M, Zawadzki RJ (2017). Review of adaptive optics OCT (AO-OCT): Principles and applications for retinal imaging [Invited]. Biomed. Opt. Express.

[11] Salas M (2016). Visualization of micro-capillaries using optical coherence tomography angiography with and without adaptive optics. Biomed. Opt. Express.

[12] Polans J (2017). Enhanced visualization of peripheral retinal vasculature with wavefront sensorless adaptive optics optical coherence tomography angiography in diabetic patients. Opt. Lett..

[13] Shirazi MF (2022). Multi-modal and multi-scale clinical retinal imaging system with pupil and retinal tracking. Sci. Rep..

[14] Palochak CMA (2019). Retinal blood velocity and flow in early diabetes and diabetic retinopathy using adaptive optics scanning laser ophthalmoscopy. J. Clin. Med..

[15] Karst SG (2018). Characterization of in vivo retinal lesions of diabetic retinopathy using adaptive optics scanning laser ophthalmoscopy. Int. J. Endocrinol..

[16] Chui TYP (2016). Longitudinal imaging of microvascular remodelling in proliferative diabetic retinopathy using adaptive optics scanning light ophthalmoscopy. Ophthalmic Physiol. Opt..

[17] Tam J (2012). Subclinical capillary changes in non-proliferative diabetic retinopathy. Optom. Vis. Sci..

[18] Ashton N (1963). Studies of the retinal capillaries in relation to diabetic and other retinopathies. Br. J. Ophthalmol..

[19] Stitt AW, Gardiner TA, Archer DB (1995). Histological and ultrastructural investigation of retinal microaneurysm development in diabetic patients. Br. J. Ophthalmol..

[20] Joussen AM (2007). Retinal Vascular Disease.

[21] Nanegrungsunk O, Patikulsila D, Sadda SR (2022). Ophthalmic imaging in diabetic retinopathy: A review. Clin. Exp. Ophthalmol..

[22] Arichika S (2014). Retinal hemorheologic characterization of early-stage diabetic retinopathy using adaptive optics scanning laser ophthalmoscopy. Investig. Ophthalmol. Vis. Sci..

[23] Sawada O (2018). Comparison between wide-angle OCT angiography and ultra-wide field fluorescein angiography for detecting non-perfusion areas and retinal neovascularization in eyes with diabetic retinopathy. Graefe’s Arch. Clin. Exp. Ophthalmol..

[24] Jia Y (2012). Split-spectrum amplitude-decorrelation angiography with optical coherence tomography. Opt. Express.

[25] Spaide RF, Fujimoto JG, Waheed NK, Sadda SR, Staurenghi G (2018). Optical coherence tomography angiography. Prog. Retin. Eye Res..

[26] Klein S, Staring M, Murphy K, Viergever MA, Pluim JPW (2010). Elastix: A toolbox for intensity-based medical image registration. IEEE Trans. Med. Imaging.

[27] Willerslev A, Li XQ, Cordtz P, Munch IC, Larsen M (2014). Retinal and choroidal intravascular spectral-domain optical coherence tomography. Acta Ophthalmol..

[28] Willerslev A, Li XQ, Munch IC, Larsen M (2014). Flow patterns on spectral-domain optical coherence tomography reveal flow directions at retinal vessel bifurcations. Acta Ophthalmol..

[29] Seidel G (2016). Estimating retinal blood flow velocities by optical coherence tomography. JAMA Ophthalmol..

[30] Kappelgaard P, Holfort SK, Klefter ON, Larsen M (2018). Retinal vessel diameter changes in relation to dark adaptation and acute hyperglycemia. J. Ophthalmol..

[31] Zhou DB (2021). Quantification of intermittent retinal capillary perfusion in sickle cell disease. Biomed. Opt. Express.

[32] Pinhas A (2022). Insights into sickle cell disease through the retinal microvasculature: Adaptive optics scanning light ophthalmoscopy correlates of clinical OCT angiography. Ophthalmol. Sci..

[33] Bek T, Ledet T (1996). Vascular occlusion in diabetic retinopathy. A qualitative and quantitative histopathological study. Acta Ophthalmol. Scand..

[34] Bek T (2002). A clinicopathological study of venous loops and reduplications in diabetic retinopathy. Acta Ophthalmol. Scand..

[35] An D, Tan B, Yu D, Balaratnasingam C (2022). Differentiating microaneurysm pathophysiology in diabetic retinopathy through objective analysis of capillary nonperfusion, inflammation, and pericytes. Diabetes.

[36] Balaratnasingam, C., An, D., Hein, M., Yu, P. & Yu, D.-Y. Studies of the retinal microcirculation using human donor eyes and high-resolution clinical imaging: Insights gained to guide future research in diabetic retinopathy. *Prog. Retin. Eye Res.* **94**, 101134 10.1016/j.preteyeres.2022.101134 (2023). 10.1016/j.preteyeres.2022.101134 37154065

[37] Bonnin S (2015). New insight into the macular deep vascular plexus imaged by optical coherence tomography angiography. Retina.

[38] Torm MEW (2022). Characterization of hyperreflective dots by structural and angiographic optical coherence tomography in patients with diabetic retinopathy and healthy subjects. J. Clin. Med..

[39] Vujosevic S (2020). Screening for diabetic retinopathy: New perspectives and challenges. Lancet Diabetes Endocrinol..

