# Supplementary material for: Detection of capillary abnormalities in early diabetic retinopathy using scanning laser ophthalmoscopy and optical coherence tomography combined with adaptive optics
Source: Sci Rep. 2024 Jun 11;14:13450. doi: 10.1038/s41598-024-63749-7 (PMC11166634; doi:10.1038/s41598-024-63749-7)
Supplement: Supplementary file 1 — Supplementary Table S1. [file 41598_2024_63749_MOESM1_ESM.docx]

**Supplementary Information**

**Supplementary Table S1.** Image/scan locations of AO-SLO, -OCT, and -OCTA used for the participants in Table 1.

| **Parti-cipant** | **AO-SLO image location** | **AO-OCT scan location** | **AO-OCTA scan location** |
| --- | --- | --- | --- |
| 1 | OD: 0.8° N; 1.6° I x3, 1.9° T; 1.6° S  OS: 0.7° N; 1.5° I, 5.2° T; 5.6° I | OD: 0.5° T; 1.9° I, 1.9° T; 1.6° S | OD: 0° T; 0° S  OS: 1.2° N; 1° I |
| 2 | OD: 0.1° T; 4.3° S, 0.2° N; 1.8° S x3, 3.3° T; 2.5° I, 9.6° T; 0.9° I  OS: 0.5° T; 3.1° S, 0.6° T; 1.6° S, 0.7° N; 2.9° S, 1.2° T; 1.7° S x2, 1.9° N; 3.7° S, 13.8° T; 5.9° I | - | OD: 0°;0°, 2° N; 0°, 2° T; 0°  OS: 0°;0°, 2° N; 0° |
| 3 | OD: 1.2° N; 1.9° S x2  OS: 1.2° T; 1.9° S | OD: 1.2° N; 1.9° S  OS: 1.2° T; 1.9° S | - |
| 4 | OD: 2° N; 0° S, 0°;0°, 2° T; 0° S, 0°; 2° S, 0°; 2° I | OD: 0°; 2° I, 2° N; 0° S | OD:0°; 2° I, 2° N; 0° S |
| 5 | OU: 2° N; 0° S, 0°;0°, 2° T; 0° S, 0°; 2° S, 0°; 2° I | OU : 2° N;0° S, 0°;0°, 2° T; 0° S | OD: 2° N; 0° S, 0°;0°, 2° T; 0° S |
| 6 | OD: 1.6° T; 0.8° S, 0.2° N; 2.5° I, 1.6° T; 0.8° S, 1° N; 2.6° I, 2.2° N; 1.7° S  OS: 4.2° T; 0.4° I x3, 3.2° T; 0.8° S x2 | OD: 3.5° T; 0.1° S | OD: 0°;0°, 2° N;0°, 2° T; 0°  OS: 0°;0°, 2° N; 0°, 2° T; 0° |
| 7 | OD: 0.5° T; 4.8° I, 3.9° T; 11.6° S  OS: 3.7° T; 2.2° I, 0.8° N; 2.2° I x2, 2.3° N; 1° S, 2.8° T; 0.4° S, 3.7° T; 2.2° I, 5.7° T; 2.7° I | OD: 0.9° T; 0.8° S | OD: 0°;0°, 2° N; 0°, 2° T; 0° |
| 8 | OD: 0.6° N; 0.4° I, 1.2° T; 0.3° I x4, 2° N; 1.8° I, 3.9° N; 6.2° I x3, 9° N; 4° I x4, 12.8° T; 9.2° S  OS: 0.4° N; 1.5° I, 0° N; 0° I x6, 2.3° T; 11.8° S, 9.4° N; 12.6° S x3 | - | OD: 0°;0°, 2° N; 0°, 2° T; 0°  OS: 0°;0°, 2° N; 0°, 2° T; 0° |
| 9 | OD: 1.6° N; 2.2° I x2 + 2° N; 0° x3, 4.2° T; 1.8° S  OS: 1.9° N; 0° x5, 3.8° N; 0.2° S | OD: 2° N; 0° S | OD: 0°;0°, 2° N; 0°, 2° T; 0°  OS: 0°;0°, 2° N; 0°, 2° T; 0° |
| 10 | OU: 2° N; 0° S, 0°;0°, 2° T; 0° S | OD: 2° N; 0° S | OD: 2° N; 0° S |
| 11 | OU: 2° N; 0° S, 0°;0°, 2° T; 0° S | OD: 2° N; 0° S | OD: 2° N; 0° S |
| 12 | OD: 4° N; 0° S | OD: 4° N; 0° S | OD: 4° N; 0° S |
| 13 | OD: 4° N; 0° S | OD: 4° N; 0° S | OD: 4° N; 0° S |
| 14 | OD: 1.2° T; 1.4° S | OD: 6.2° T; 0.1° S | - |
| 15 | OD: 0° T; 0° S, 6.1° T; 0.3° S | OD: 6.1° T; 0.3° S | - |
| 16 | OD: 0.6° T; 2.2° S, 2.7° T; 6.2° S, 9.9° N; 11.1° S  OS: 0.1° T; 2.1° S, 10.6° N; 10.4° S | - | OD: 0° T; 0° S  OS: 0° T; 0° S |
| 17 | OD: 0.2° N; 3.2° S, 11.4° N, 9.1° S  OS: 0.2° N; 1.6° S, 10.6° N, 9.4° S | - | OS: 1.3° T; 1.6° S |
| 18 | OD: 2° N; 0° S, 0°;0° | - | OD: 2° N; 0° S, 0°;0° |
| 19 | OS: 2° N; 0° S, 0°;0°, 2° T; 0° S | - | OS: 2° N; 0° S, 0°;0°, 2° T; 0° S |
| 20 | OD: 2° N; 0° S, 0°;0°, 2° T; 0° S | - | OD: 2° N; 0° S, 0°;0°, 2° T; 0° S |
| 21 | OU: 2° N; 0° S, 0°;0°, 2° T; 0° S | - | OU: 2° N; 0° S, 0°;0°, 2° T; 0° S |

**Abbreviations:** OCT = optical coherence tomography, OCTA = optical coherence tomography angiography, AO = adaptive optics, SLO = scanning laser ophthalmoscopy, N = nasally, T = temporally, S = superiorly, I = inferiorly, ° = degree(s), – = not used. All coordinates are in relation to the center of the fovea (0°,0°). Coordinates in green indicate images used in the figures.
